# Quantifying Assemblage Turnover and Species Contributions at Ecologic Boundaries

**DOI:** 10.1371/journal.pone.0074999

**Published:** 2013-10-09

**Authors:** Lee-Ann C. Hayek, Brent Wilson

**Affiliations:** 1 Smithsonian Institution Mathematics and Statistics NMNH MRC-121, Washington D.C., United States of America; 2 Petroleum Geoscience Programme, Department of Chemical Engineering, The University of the West Indies, Saint Augustine, Trinidad, Trinidad and Tobago; Plymouth University, United Kingdom

## Abstract

Not all boundaries, whether stratigraphical or geographical, are marked by species-level changes in community composition. For example, paleodata for some sites do not show readily discernible glacial-interglacial contrasts. Rather, the proportional abundances of species can vary subtly between glacials and interglacials. This paper presents a simple quantitative measure of assemblage turnover (assemblage turnover index, ATI) that uses changes in species' proportional abundances to identify intervals of community change. A second, functionally-related index (conditioned-on-boundary index, CoBI) identifies species contributions to the total assemblage turnover. With these measures we examine benthonic foraminiferal assemblages to assess glacial/interglacial contrasts at abyssal depths. Our results indicate that these measures, ATI and CoBI, have potential as sequence stratigraphic tools in abyssal depth deposits. Many peaks in the set of values of ATI coincide with terminations at the end of glaciations and delineate peak-bounded ATI intervals (PATIs) separated by boundaries that approximate to glacial terminations and to transgressions at neritic depths. These measures, however, can be used to evaluate the assemblage turnover and composition at any defined ecological or paleoecological boundary. The section used is from Ocean Drilling Program (OPD) Hole 994C, drilled on the Blake Ridge, offshore SE USA.

## Introduction

Biostratigraphers historically have sought means to subdivide the sedimentary stratigraphic record as finely as possible. There is, however, evidently a limit to the zonal resolution that can be attained using a single fossil group [1]. For example, the majority of the Pleistocene, the base of which is placed at 2.588 Ma [2], is currently ascribed to the *Globorotalia truncatulinoides truncatulinoides* (dOrbigny) planktonic foraminiferal Zone [3] or to Zones PT1a and PTIb based on planktonic foraminifera [4] (author names are given at the first mention of any species).

It has long been appreciated, however, that glacial and interglacial fauna and flora within the Pleistocene differ [5], both on land [6] and in the oceans [7]. In Chile, for example, plant leaf architecture changes from a mixture of species belonging to the cool temperate North Patagonian Forest and more thermophilous rain forest vegetation between glacials and interglacials [8]. Glacial-interglacial contrasts in the insect community have been recorded in Greenland [9]. Such changes have been recorded among some foraminifera, but not at all sites. Schott [10] found that *Globorotalia menardii* (d'Orbigny) in the Indian Ocean was more abundant in interglacial than in glacial sediments. Phleger et al. [11], in a study of North Atlantic foraminifera, noted the presence in piston cores of “layers of faunas normal for their latitude alternating with faunas typical of a higher latitude,” while Bandy [12] was able to distinguish glacials from interglacials off southern California using the ratio between populations of sinistrally and dextrally coiled *Neogloboquadrina pachyderma* (Ehrenberg). Streeter [13] found that Atlantic benthonic foraminifera at depths >2500 m varied greatly over the last 150 ka and suggested that this arose because of depression and elevation of faunas through a depth range of several hundred meters between glacials and interglacials [14]. Streeter and Lavery [15] recorded that uppermost Pleistocene faunas in cores from the western North Atlantic slope and rise north of 35°N were dominated by *Uvigerina*, but that *Hoeglundina* dominated in the Holocene. This faunal transition was apparently diachronous, occurring at ∼12 ka at 3,000 m, but at ∼8 ka at 4,000 m. Thomas et al. [16] examined benthonic foraminifera in two lower bathyal (∼1700 m) and abyssal (∼3500 m) piston cores spanning the last 45 ka in the northeastern Atlantic Ocean. They found that *Epistominella exigua* (Brady) and *Alabaminella weddellensis* (Earland), which bloom opportunistically where a spring plankton bloom produces a pulse of phytodetritus, were rare during the last glacial maximum but abundant in the Holocene. In contrast, *Cassidulina*, *Pullenia*, bolivinids, buliminids and uvigerinids were common during glacial MIS (marine isotope stages) 2, 3 and 4, although the interglacial MIS 3 was not as warm as other Late Quaternary interglacials [17]. Thomas et al. [16] suggested that this reflects an enhanced organic carbon flux during glacials, rather than sluggish glacial bottom circulation leading to poorly oxygenated bottom water. This may be related to the plankton multiplier effect proposed by Woods and Barkmann [18], in which a diminished greenhouse effect during glacials reduces radiative forcing of the ocean, increasing the depth of winter convection. This in turn increases the annual resupply of nutrients to the euphotic zone, which leads to increased annual primary production. Gaby and Sen Gupta [19] found glacial and postglacial assemblages of the abyssal Venezuela Basin to differ, the Holocene fauna containing abundant *Cibicides wuellerstorfi* (Schwager), *Melonis pompilioides* (Fichtel and Moll), *Nuttallides umbonifera* (Cushman), and *Pullenia* sp., while the fauna in the last glacial was dominated by *Massilina* sp., *Globocassidulina subglobosa* (Brady) and *Nummoloculina irregularis* (d'Orbigny).

Glacial-interglacial contrasts in the benthonic foraminiferal fauna are not everywhere marked, however. Streeter and Lavery [15] wrote that on the western North Atlantic continental rise below 4,000 m, “the glacial to modern faunal shift is subtle, but it clearly occurs later than on the upper rise.” Sen Gupta et al. [20] examined benthonic foraminifera over the past 127 ka in three bathyal cores (depths near 2000 m) from the western Grenada Basin, eastern Caribbean Sea. They found only subtle changes, rather than drastic turnovers, at glacial-interglacial boundaries based on the abundance of *Globorotalia menardii*. They stated that neither species richness S nor the information function 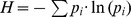, where p_i_ is the proportional abundance of the *i*th species) showed any distinct stratigraphic trend (although H is not expected to show such a trend [21]). However, they suggested *Nuttallides umbonifera*, *Bulimina buchiana* dOrbigny and *Chilostomella oolina* Schwager to be rarer in the last glacial than in the two bounding glacials. Wilson [22], [23] examined the benthonic foraminifera in two bathyal piston cores near the northern Leeward Islands, eastern Caribbean Sea. He did not find any marked faunal changes at the Pleistocene-Holocene boundary, but showed that the organic flux in one core decreased gradually through the entire core. Wilson [24] found only weak evidence of Milankovich cycles in the Upper Quaternary of ODP Hole 1006A (Santaren Channel, offshore western Bahamas), where *Globocassidulina subglobosa* and *Cibicidoides* aff. *C. io* (Cushman) were smaller assemblage components during most glacial MISs. However, the percentages of these species varied between odd-numbered MISs and they were insignificantly correlated with one another, *G. subglobosa* being rare in MIS 9 while *C*. aff. *C. io* was common.

**Figure 1:**
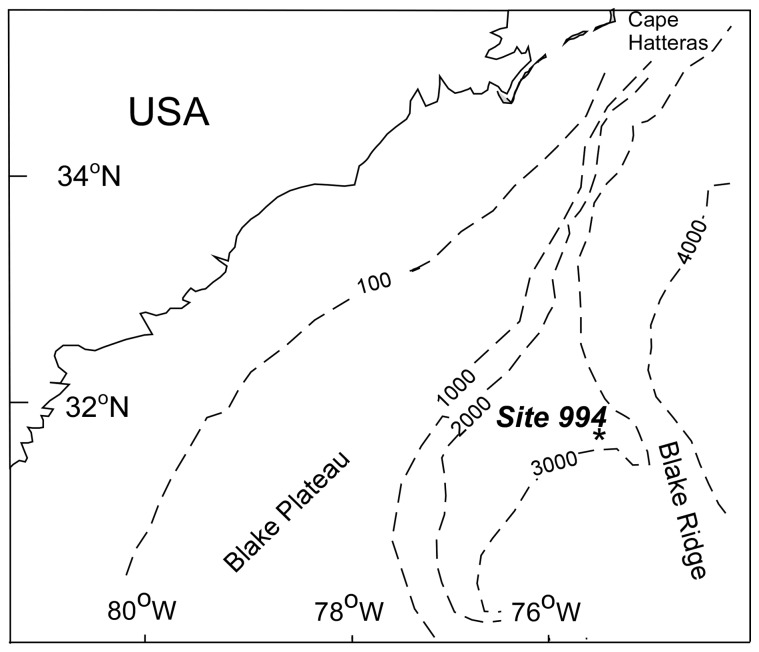
The location of ODP Hole 994C, Blake Ridge.

The inability to detect glacial-interglacial contrasts at all sites appears to arise because not all sites show marked changes in community composition at the species level at glacial-interglacial boundaries. Rather, the proportional abundances of species vary between glacials and interglacials to differing degrees. This paper presents a simple quantitative measure, the assemblage turnover index (ATI), which uses changes in species' proportional abundances to identify intervals of marked community change. Whittaker [25] distinguished two categories of diversity: inventory diversity, which calculates the diversity of associations within samples (point diversity) or habitats (α diversity); and differentiation diversity, which examines the change in diversity between samples (pattern diversity) or habitats (β diversity). The assemblage turnover index presented here is a form of differentiation diversity. A conditioned-on-boundary index (CoBI), developed as a function of the ATI, identifies species that contribute most to maxima and minima in sets of values of the ATI. The section used in this demonstration is from Ocean Drilling Program (OPD) Hole 994C, drilled on the Blake Ridge, offshore SE USA (Figure 1). Although Bhaumik and Gupta [26], [27] and Mohan et al. [28] have examined Neogene benthonic foraminifera from this and nearby ODP Holes, glacial-interglacial contrasts have not been recorded at the Blake Ridge before this study.

## Site Description

ODP Site 994 (31°47.139′N, 75°32.753′W; water depth 2799 m) is situated on the sediment drift deposit that forms Blake Ridge [29], [30], a drift deposit consisting of current-lain sediment east of the Blake Plateau. The Blake Ridge was deposited by the Western Boundary Undercurrent, this being a thermohaline-induced contour current (a current that flows parallel to bathymetric contours), which flows southward along the U.S. continental margin [31]. The Western Boundary Undercurrent transported clays eroded from eastern North America north of 40°N, at least as far south as Puerto Rico [32]. ODP Hole 994C cored a 700 m-thick succession of clays with calcareous nannofossils within which there are no obvious depositional hiatuses [30]. In this paper we examine the topmost ∼14 m of sediment in ODP Hole 994C. Mass sediment transport complexes are absent. The studied section, which is a distinct lithostratigraphic unit termed Unit 1 [30], consists of light gray to gray and greenish gray nannofossil-rich clays in beds up to 1.20 m thick. The presence of the trace fossil *Zoophycos* indicates that some bioturbation has taken place, but mostly at bedding planes. Biostratigraphic correlation within the Quaternary of ODP Hole 994C is limited. Okada [33] found the first occurrence of *Emiliania huxleyi* (Lohmann) at 8.05–9.05 meters below the seafloor (mbsf), between OPD Hole 994C Core 2H-3, 65 cm and 2H-4, 15 cm, for which he suggested an age of 0.26 mya. This indicates a depositional rate of 3.5 cm/ka in the uppermost part of the Hole. The first appearance of *E. huxleyi* has subsequently been placed between 0.262–0.264 Myr [34].

Bhaumik and Gupta [27] studied the benthonic foraminifera in nearby ODP Hole 997A, and found *Brizalina paula* (Cushman and Cahill), *Cibicidoides kullenbergi* (Parker), *Uvigerina hispidocostata* Cushman and Todd and *Uvigerina peregrina* Cushman to be abundant in that part of the section within the gas hydrate zone. Bhaumik and Gupta [26] examined benthic foraminiferal assemblages (>125 mm size fraction), the information function H and total organic carbon in Hole 997A during the late Neogene (last 5.4 Ma). They concluded that *B. paula, Cassidulina carinata* Silvestri, *Chilostomella oolina, Fursekoina fusiformis* (Cushman), *Globobulimina pacifica* Cushman, *Nonionella auris* (d'Orbigny) and *Trifarina bradyi* are potential methane seep-related foraminifera, while *U. hispidocostata, U. peregrina, U. proboscidea* (Schwager) and *Melonis barleeanus* (Williamson) indicate a high organic carbon flux independent of deep-sea oxygenation.

## Materials and Methods

Sixty seven samples of 20 cm^3^ were taken at 20 cm intervals from ODP Hole 994C, Cores 1 and 2, between 0.08–13.25 mbsf. They were provided by the Ocean Drilling Program (ODP) that is sponsored by the U.S. National Science Foundation (NSF) and participating countries. The cored site being in international waters, no specific permissions were required for these locations/activities. The material comprising fossils, sampling did not involve endangered or protected species. Each sample was ∼2 cm thick and represents ∼600 years. Samples were soaked in water until disaggregated, washed over a 63 µm mesh to remove silt and clay, and dried over a gentle heat. An attempt was made to pick N = 250–300 specimens of benthonic foraminifera from the >63 µm fraction from each sample. However, only 42 samples yielded >250 specimens (mean, 251 specimens per sample, minimum 104). The methods on assemblages used here have been reported by Wilson [35]. The foraminifera were sorted into species and identified using Cushman [36], [37], [38], [39], [40], [41], [42], Cushman and Henbest [43], Phleger and Parker [44], Phleger et al. [11], Parker [45], Pflum and Frericks [46] and Mohan et al. [28]. The number of specimens (n_i_) was recorded for each species or species group (i.e., rare species in the same genus that were left in open nomenclature and grouped together).

**Figure 2:**
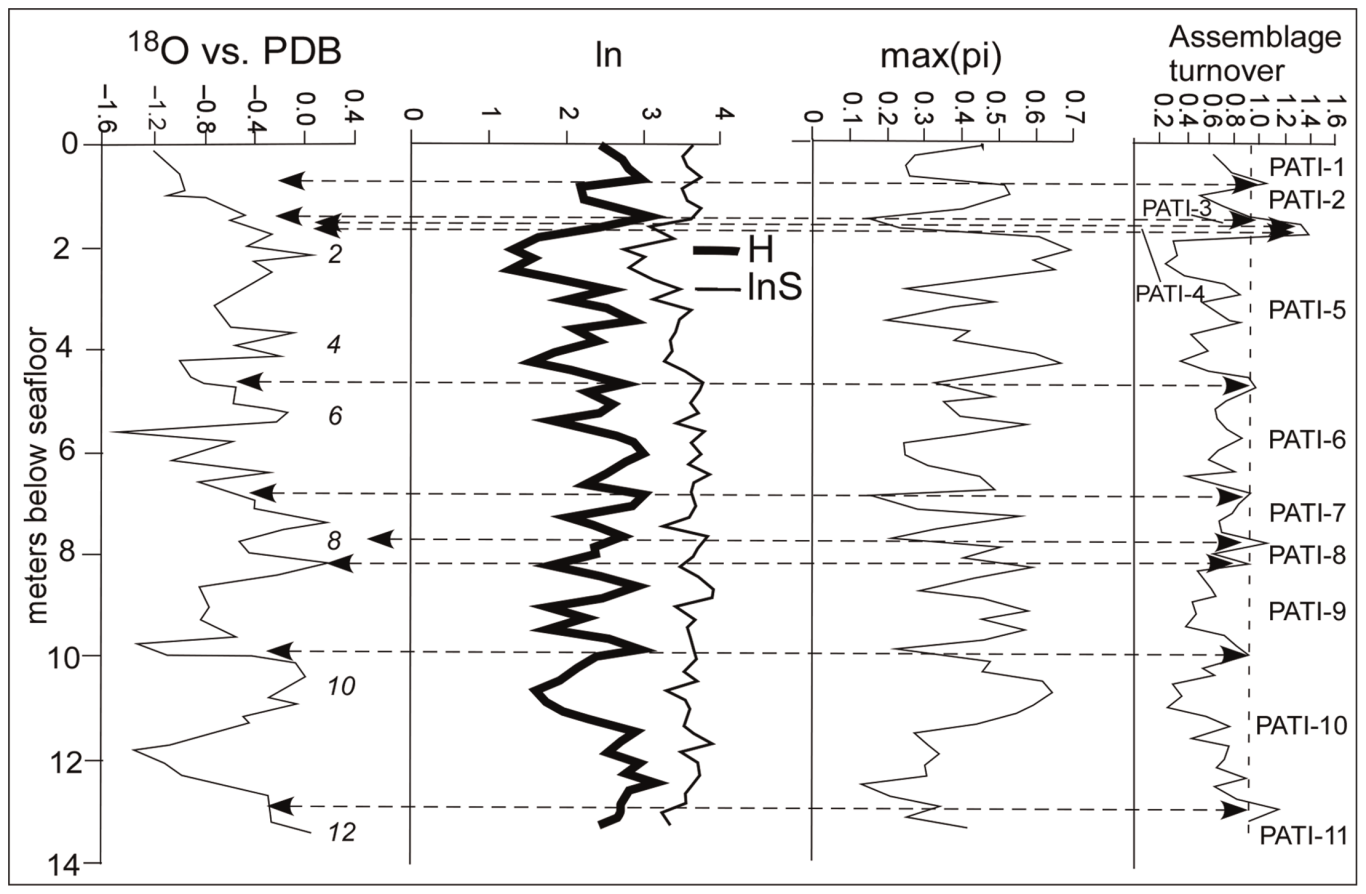
δ18O from Oba et. [53], the information function H, species richness S (graphed as lnS to facilitate comparison with H), max(p_i_) and between sample assemblage turnover index (ATI_s_) [from this paper] in the Upper Quaternary, ODP Hole 994C, Blake Ridge. Numbers in italics indicate marine isotope stages; PATI are peak-bounded ATI_s_ intervals.

*Elphidium excavatum* (Terquem), *Epistominella takayanagii* Iwasa, *Quinqueloculina poeyana* d'Orbigny and *Quinqueloculina* ex gr. *lamarckiana* d'Orbigny, which are typical of neritic water, were regarded as allochthonous and excluded from this analysis. *Elphidium* is typically regarded as a shallow-water genus [47] that has to be removed from data sets of studies of bathyal foraminifera [48]. Sen Gupta and others [49] recorded abundant *E. excavatum* on the Louisiana continental shelf. In the southern North Sea, it dominates the foraminiferal fauna at depths between 25–30 m [50]. *Epistominella takayanagii* has been recorded from Chaleur Bay eastern Canada, mostly in waters <100 m deep [51], and may have been transported southwards to ODP Site 994C. The proportional abundance of both *E. excavatum* and *E. takayanagii* peaked in MIS 10, glacial cycle E, as did the percentage of overall allochthonous, shallow-water species. A single specimen of *Stilostomella lepidula* (Schwager) recovered from 5.45 mbsf was presumed to be reworked, this species having gone extinct during middle Pleistocene times [52], and was excluded from further analysis. This left a presumed predominantly in situ abyssal assemblage, within which there may have been some slight downslope transport of *Angulogerina occidentalis* (Cushman), *Bulimina aculeata* d'Orbigny, *B. alazanensis* Cushman, *Cibicides* sp., *Fursenkoina fusiformis* (Williamson), *Globocassidulina obtusa* (Williamson) and *Nuttallides rugosa* (Phleger and Parker) [35]. This presumed in situ assemblage forms the subject of the remainder of this paper. To examine turnover of an entire assemblage quantitatively across a delineated boundary we developed the ATI index. For a set of samples from a given site, the Assemblage Turnover Index for each pair of adjacent samples is defined as
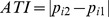
(1)


 in which p_i1_ and p_i2_ are the proportional abundances of the *i*th species, i = 1,…, s, in the lower and upper samples (see Appendix S1 for a glossary of terms). This assemblage turnover index between samples will be denoted as ATI_s_. Note that although for each sample 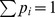, the measure ATI_s_ can be >1. Thus, ATI_s_ gives the proportion or percent of turnover or change specifically across a defined or particular boundary. We calculated the mean 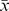, and standard deviation, σ, of values of ATI_s_ over all samples within the core. To develop our control chart we determined all points with 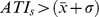, which were then deemed to be positions of major turnover. Oba et al. [53] presented a δ18O curve for ODP Hole 994C (see Figure 2). Their samples were taken at irregular intervals (sample spacing 7–49 cm; mean 22.6 cm, sd 10.9 cm). The values of δ18O for the samples used here were interpolated from Oba et al. 's [53] curve and correlation between ATI_s_ and interpolated δ18O was calculated. Because Oba et al. 's [53] uppermost sample was taken at 0.14 mbsf, it was not possible to estimate the δ18O value for the uppermost sample picked for this study. Point (sample) values of species richness S and the information function H were calculated. Dominance was determined using max(p_i_), the proportional abundance of the most abundant species in each sample [54]. We chose to calculate correlations between ATI_s_, S, H and max(p_i_) using the upper (younger) sample in each sample pair.

Peaks in ATI_s_, those values larger than our designated control value of 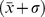, were used to divide the succession into peak-bounded ATI_s_ (PATI-) intervals. These intervals were numbered, commencing from PATI-1 for the most recent. PATI-1 and the oldest PATI are incomplete, their upper and lower boundaries respectively not being bounded by ATI_s_ peaks.

To assess which species contributed most to the ATI at the PATI boundaries, a conditioned –on-boundary index CoBI was derived. CoBI provides the proportion that each species within an assemblage contributed to the change or turnover specifically across the PATI boundary. For each species at any PATI boundary
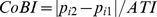
(2)


where p_ij_, j = 1,2 are the *i*th species proportions on either side of the selected boundary of interest and at which the ATI is calculated.

There are two forms of CoBI:

1. Partial conditioned-on-boundary index, CoBI_p_, in which the assemblage turnover index ATI_s_ was calculated between the entire set of samples within the PATI below the ATI peak and the first sample immediately above the peak. In this case, the ATI is designated as ATI_p_. The value of ATI ( =  ATI_p_) was substituted into equation (2), as were p_i1_, the proportional abundance of the *i*th species in the entire PATI below the peak in ATI_p_, and p_i2_, the proportional abundance of that *i*th species in the first sample above the peak in ATI_s._ The proportional contribution of each species to ATI_p_ was assessed from the vector of CoBI_p_ values at each ATI_s_ peak.
2. Thorough conditioned-on-boundary index CoBI_t_, in which the ATI is denoted as ATI_t_, was calculated between the values in two complete PATIs separated by the peak in ATI_s_ (see Figure 2). The value of ATI_t_ was substituted into equation (2), as were p_i1_ and p_i2_, the proportional abundance of the *i*th species in the two PATIs separated by the peak in ATI_t_. The proportional contribution of each species to the value of ATI_t_ was assessed from the vector of partial CoBI for each ATI_s_ peak.

Thus, to detect change between the total set of samples from the assemblages within two distinct PATIs we evaluate the ATI  =  ATI_t_ at the boundary between these two. The partial indices are used to detect assemblage change exactly at the boundary between a single PATI and the next contiguous sample.

## Results

### Assemblage Turnover Index (ATI = ATI_s_) Between Samples

**Figure 3:**
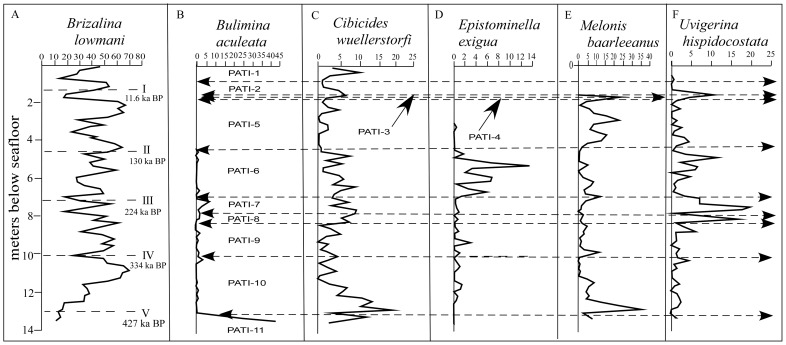
Percentage abundances of selected species in the Upper Quaternary of ODP Hole 994C, species presented in alphabetical order. A. *Bulimina aculeata*. B. *Brizalina lowmani*. C. *Cibicides wuellerstorfi*. D. *Epistominella exigua*. E. *Melonis baarleeanus*. F. *Uvigerina hispidocostata*. Dashed lines in A indicate positions of Terminations I – V, with ages in ka BP. Dashed lines in B – F indicate boundaries between PATIs 1 to 11. Terminations are indicated by Roman numerals in order of increasing age.

Our presumed in situ, abyssal fauna comprised 16,184 specimens in 157 species (see File S1), and was dominated by *Brizalina lowmani* (Phleger and Parker) (42.1% of total recovery; range 6.9–70.0% per sample) with subdominant *Globocassidulina obtusa* (Williamson) (8.9% of total recovery; range 0–21.9% per sample). Thirteen other species formed >1% of total recovery: *Bulimina aculeata* d'Orbigny, *Cassidulina laevigata* d'Orbigny, *Cibicides wuellerstorfi*, *Cibicidoides robertsonianus* (Brady), *Epistominella exigua*, *Gyroidina lamarckiana* (d'Orbigny), *Hoeglundina elegans* (d'Orbigny), *Melonis baarleeanus* (Williamson), *M. pompilioides* (Fichtel and Moll), *Oridorsalis umbonatus* (Reuss), *Pullenia bulloides* (d'Orbigny), *Pyrgo lucernula* (Schwager) and *Uvigerina hispidocostata* Cushman and Todd. The distributions of selected species are shown in Figure 3.

The assemblage turnover index between adjacent samples ranged from ATI_s_  = 0.263–1.421(x¯ = 0.710, σ = 0.233) (Figure 2), indicating total assemblage change from 26% to 142%. The value of ATI_s_ exceeded x¯+ σ = 0.943 across nine pairs of samples. We chose to include the borderline value of ATI_s_ at 9.85 mbsf, where it nevertheless formed a pronounced peak. We computed correlations of ATI with the indices H and max (p_i_) and with δ18O. Although the formulae for these measures utilize the relative abundances, there is no linear functional relationship among them that necessitates a significant correlation. The ATI_s_ was positively correlated with the information function H for the younger sample in the pair (r = 0.62, p<0.0001). ATI_s_ was negatively correlated with max(p_i_) (r = –0.65, p<0.0001), which indicates a change in dominance across peaks in ATI_s_, and negatively correlated with δ18O (r = –0.32, p<0.01). H and δ18O were in turn significantly negatively correlated (r = –0.53, p<0.0001).

### Partial Conditioned-on-Boundary Index (CoBI_p_)

The accepted peaks in the ATI_s_ delimited eleven peak-bounded ATI intervals (PATI-). These intervals contained between one (PATI-3 and PATI-4) and fifteen (PATI-5 and PATI-10) samples. The ATI_p_ used for CoBI_p_ ranged between 0.83–1.33 (PATI-6/5 and PATI-4/3 boundaries respectively; Table 1), which indicates assemblage turnover between 83% and 133% at the boundaries. Only 31 species showed CoBI_p_ >0.02 (i.e., accounted for >2% of ATI_p_) at any one PATI boundary (Table 1) and these species differed over the PATI boundaries.

**Table 1:** Partial conditioned-on-boundary index (CoBI_p_) for peak-bounded ATI_s_ (PATI-) intervals in the Upper Quaternary in ODP Hole 994C, Blake Ridge.

Boundary	PATI-11/10	PATI-10/9	PATI-9/8	PATI-8/7	PATI-7/6	PATI-6/5	PATI-5/4	PATI-4/3	PATI-3/2	PATI-2/1
ATI	1.09	0.89	0.88	0.93	0.91	0.83	0.96	1.33	0.95	0.97
*Alabaminella weddellensis*	-	-	-	-	-	-	-	-	-	0.02
*Brizalina lowmani*	-	**0.20**	**0.35**	***0.33***	**0.47**	**0.27**	**0.08**	-	**0.26**	***0.21***
*Bulimina aculeata*	*0.31*	-	-	-	-	-	-	-	-	-
*Cassidulina laevigata*	-	0.02	-	-	0.04	-	**0.09**	0.03	*0.04*	-
*Cibicides wuellerstorfi*	*0.03*	**0.05**	**0.09**	0.03	-	**0.09**	-	0.04	0.02	-
*Cibicidoides bradyi*	-	-	-	-	-	-	0.04	0.03	-	0.03
*Cibicidoides robertsonianus*	-	-	-	-	-	0.03	-	0.02	0.03	-
*Epistominella exigua*	-	-	-	-	**0.06**	-	-	-	-	-
*Eponides regularis*	-	-	-	-	-	-	-	-	-	***0.10***
*Gavelinopsis praegeri*	-	-	-	-	-	-	-	-	-	0.03
*Globobulimina pacifica*	-	-	-	-	-	-	-	0.03	0.03	-
*Globocassidulina murrhina*	-	-	-	-	-	-	-	0.04	-	*0.02*
*Globocassidulina obtusa*	0.03	0.03	0.02	-	**0.09**	-	-	-	-	-
*Globocassidulina subglobosa*	-	0.04	-	-	-	-	**0.07**	**0.05**	-	-
*Gyroidina lamarckiana*	-	**0.13**	-	-	-	**0.05**	-	0.03	-	**0.22**
*Gyroidinoides soldanii*	-	0.03	-	-	-	-	-	-	-	-
*Hoeglundina elegans*	-	-	-	-	-	**0.15**	-	-	-	0.03
*Martinottiella communis*	-	-	-	-	-	-	**0.17**	***0.12***	-	-
*Melonis baarleeanus*	**0.27**	**0.11**	-	*0.02*	0.02	-	**0.24**	***0.17***	-	-
*Melonis pompilioides*	0.04	-	-	**0.21**	***0.06***	-	-	-	**0.08**	*0.03*
*Nonionella bradyi*	-	-	-	-	-	0.02	-	-	-	-
*Oridorsalis umbonatus*	-	**0.08**	**0.05**	-	-	-	-	-	-	-
*Pullenia bulloides*	-	0.03	0.04	-	-	-	0.02	-	-	-
*Pullenia* spp.	-	-	-	-	-	-	-	0.03	*0.03*	-
*Pyrgo lucernula*	-	-	**0.05**	*0.03*	-	-	0.03	-	*0.03*	-
*Pyrgo murrhina*	-	0.03	-	-	-	**0.06**	-	-	-	-
*Robertinoides bradyi*	-	-	-	-	-	0.02	-	-	-	-
*Sigmoilopsis schlumbergeri*	*0.02*	-	-	-	-	-	-	***0.08***	**0.09**	-
*Trifarina angulosa*	-	-	-	-	-	0.02	-	-	-	0.03
*Uvigerina celtica*	-	-	0.03	*0.04*	-	-	**0.07**	0.04	-	-
*Uvigerina hispidocostata*	-	-	**0.18**	**0.09**	*0.04*	0.02	0.04	**0.05**	***0.11***	-

Dashes indicate CoBI_p_ <0.02, bold indicates CoBI_p_ >0.05. Italics indicate that a species' proportional abundance decreased over a PATI boundary.

Thirteen species had a CoBI_p_ of 0.02–0.05 at any one boundary, while 18 (∼11.5% of all species recorded) had a CoBI_p_ >0.05 across any one PATI boundary. Seven species (*Cassidulina laevigata*, *Epistominella exigua, Eponides regularis* Phleger and Parker, *Globocassidulina obtusa*, *Hoeglundina elegans*, *Pyrgo murrhina* (Schwager), *Uvigerina celtica* Schönfeld) had a CoBI_p_ >0.05 across one PATI boundary only. Two species (*Melonis baarleeanus*, *Uvigerina hispidocostata*) had a CoBI_p_ >0.05 across four PATI boundaries. *Brizalina lowmani* fluctuated most markedly, having the CoBI_p_ >0.05 across eight PATI boundaries. The maximum CoBI_p_ was 0.47, indicating 47% change in *B. lowmani* abundance across the PATI-7/6 boundary. Four other species (*Bulimina aculeata*, *Gyroidina lamarckiana*, *Melonis baarleeanus*, *M. pompilioides*) had a maximum CoBI_p_ >0.20 across any one PATI boundary. Using CoBI_p_, *Brizalina lowmani* decreased in proportional abundance across the boundaries between PATI-8/7 and 2/1, *M. pompilioides* between PATI-7/6 and 2/1, and *U. hispidocostata* between PATI-7/6 and 3/2.

### Thorough Conditioned-on-Boundary Index (CoBI_t_)

The ATI_t_ used for CoBI_t_ ranged between 0.36–1.33 (PATI-10/9 and PATI-4/3 boundaries respectively; Table 2), or an observed total assemblage change ranging from 36% to 133% between the PATI pairs from PATI-1/2 through PATI-10/11. Thirty three species resulted in a value of CoBI_t_ >0.02 (i.e., accounted for >2% of ATI_t_) at any one PATI boundary. Twenty eight species contributed to >0.02 of both ATI_p_ and ATI_t_. Seventeen species had a CoBI_t_ of 0.02–0.05 at any one boundary. Thus, only sixteen species (∼10% of all species recorded) had a CoBI_t_ *>*0.05 across any one PATI boundary. The highest CoBI_t_ was 0.31 for *Brizalina lowmani*, indicating pronounced change in dominance across the PATI-3/2 boundary, while *Bulimina aculeata* presented a comparable CoBI_t_ of 0.29 across the PATI-11/10 boundary. All other species contributed a maximum CoBI_t_ <0.20, although the maxima for *Globocassidulina obtusa* and *Melonis baarleeanus* were 0.19 and 0.17 respectively. The CoBI_t_ for *B. lowmani* was >0.02 across eight PATI boundaries, while those for *G. obtusa* were >0.02 across six boundaries and for *M. baarleeanus* and *Cibicidoides robertsonianus* across five.

**Table 2:** Thorough conditioned-on-boundary index (CoBI_t_) for peak-bounded ATI_s_ (PATI-) intervals in the Upper Quaternary in ODP Hole 994C, Blake Ridge.

PATI boundary	PATI-11/10	PATI-10/9	PATI-9/8	PATI-8/7	PATI-7/6	PATI-6/5	PATI-5/4	PATI-4/3	PATI-3/2	PATI-2/1
ATI_t_	1.19	0.36	0.55	0.54	0.56	0.56	1.23	1.33	1.03	0.67
*Alabaminella weddellensis*	-	0.04	-	-	-	0.02	-	-	-	-
*Brizalina lowmani*	**0.30**	**0.06**	0.03	***0.28***	**0.18**	**0.17**	***0.29***	-	**0.31**	***0.29***
*Bulimina aculeata*	***0.29***	-	-	0.04	***0.05***	-	-	-	-	-
*Bulimina alazanensis*	-	*0.03*	-	-	-	-	-	-	-	-
*Bulimina striata mexicana*	-	*0.03*	-	-	-	-	-	-	-	*0.02*
*Cassidulina laevigata*	0.02	***0.05***	-	-	-	0.04	0.04	*0.03*	*0.04*	-
*Cibicides wuellerstorfi*	-	***0.08***	**0.09**	-	*0.04*	***0.05***	-	0.04	*0.04*	0.03
*Cibicidoides bradyi*	-	-	-	-	-	-	0.03	*0.03*	-	-
*Cibicidoides robertsonianus*	-	-	-	-	-	0.02	*0.02*	0.02	*0.02*	0.02
*Epistominella exigua*	-	-	-	-	**0.08**	***0.09***	-	-	-	-
*Eponides regularis*	-	-	-	-	-	-	-	-	-	**0.05**
*Gavelinopsis praegeri*	-	-	-	-	-	-	-	-	-	0.02
*Globobulimina pacifica*	-	-	-	-	-	-	-	0.03	*0.03*	-
*Globocassidulina murrhina*	-	-	-	-	0.02	*0.02*	-	0.04	*0.03*	**0.06**
*Globocassidulina obtusa*	**0.07**	**0.17**	***0.19***	**0.05**	**-**	**0.05**	***0.08***	0.02	-	-
*Globocassidulina subglobosa*	-	-	-	-	-	-	**0.05**	***0.05***	-	-
*Gyroidina lamarckiana*	-	-	*0.03*	-	**0.05**	*0.04*	-	0.03	-	**0.11**
*Gyroidinoides soldanii*	-	-	*0.02*	0.02	-	-	-	-	-	-
*Hoeglundina elegans*	-	-	-	**0.05**	*0.04*	-	-	-	0.02	*0.03*
*Lagena spp.*	-	-	-	-	-	-	-	-	0.02	*0.03*
*Martinottiella communis*	-	-	-	-	-	-	**0.13**	***0.12***	-	-
*Melonis baarleeanus*	-	**0.08**	0.02	0.03	-	**0.09**	**0.12**	***0.17***	-	-
*Melonis pompilioides*	*0.03*	*0.04*	0.03	**0.06**	***0.07***	*0.02*	-	-	0.02	-
*Nonionella bradyi*	-	-	-	-	-	-	-	-	-	0.03
*Nuttallides rugosa*	-	-	*0.03*	-	-	-	-	-	-	0.02
*Oridorsalis umbonatus*	*0.03*	0.03	-	-	-	*0.03*	*0.02*	-	-	-
*Pullenia bulloides*	*0.04*	-	0.03	-	*0.03*	-	-	-	-	-
*Pullenia quinqueloba*	-	-	-	0.03	-	-	-	-	0.02	0.02
*Pullenia spp.*	-	-	-	-	-	-	-	0.03	*0.03*	*0.02*
*Pyrgo lucernula*	-	-	0.04	*0.04*	-	0.03	-	-	*0.03*	-
*Sigmoilopsis schlumbergeri*	-	-	0.02	-	-	-	-	**0.08**	***0.07***	***0.05***
*Uvigerina celtica*	-	-	**0.06**	***0.05***	0.02	-	**0.05**	*0.04*	*0.02*	-
*Uvigerina hispidocostata*	-	0.02	**0.13**	0.03	***0.13***	*0.04*	0.02	**0.05**	***0.10***	-

Dashes indicate CoBI_t_ <0.02, bold indicates CoBI_t_ >0.05. Italics indicate that a species' proportional abundance decreased over a PATI boundary.

## Discussion

The assemblage turnover index is a form of differentiation diversity sensu Whittaker [25]. It is here presented at two scales, of which the end members are (a) ATI_s_, which corresponds to Whittaker's (1972) pattern or between-sample diversity, and (b) ATI_t_, which is here taken as corresponding to his between-habitat or β-diversity. ATI_s_ was strongly positively correlated with the information function H and negatively correlated with max(p_i_) for the younger of the samples in the sample pairs used in its calculation. This indicates that assemblage turnover – the sum of the changes in proportional abundances of species – increases with increasing diversity and with decreasing dominance (i.e. increasing equitability).

The correlation between ATI_s,_ and interpolated values of δ18O was significant and negative (Figure 2). Oxygen isotopes have been used to erect a paleotemperature record of marine isotope stages (MISs) that is reliable back to MIS 16, 650 ka [17], within which odd numbered MISs are interglacials [55] and against which faunal changes can be compared. Much of the time between MIS 1–12 (the interval examined in this study) consists of 100 ka MIS couplets [56], [57]. Broecker and Van Donk [58] grouped the MISs into glacial cycles (segments of two to four MISs) that were separated by terminations (i.e., pronounced boundaries between isotopic maxima and minima). Because warming during deglaciation occurs more rapidly than does cooling during the development of glaciation [59], each termination separates a preceding glacial from a succeeding interglacial. MIS 3 being a subdued glaciation, there is no termination between MIS 4 and 3, but Termination T-I occurs between MIS 2 and 1, and T-II between MIS 6 and 5. The interval examined during this study encompasses terminations T-V to T-I, which separated Glacial Cycles F to A (Figure 3). Cheng et al. [60] positioned terminations at the mid-point between the peaks and troughs in the graph of δ18O. However, because the δ18O curve for ODP Hole 994C presented by Oba et al. [53] was based on irregularly spaced samples their technique cannot be used here. It does appear, however, that the boundaries between PATI-11/10, 10/9, 7/6 and 6/5 and 2/1 approximate to terminations T-V through T-I, respectively. Thus, the slower onsets of glacials are marked by low levels of turnover, ATI_s_, while the more rapid transitions to interglacials are marked by peaks in ATI_s_. Because the peaks in ATI_s_ occurred within terminations, the correlation between ATI_s_ and δ18O is low.

Not all peaks in ATI_s_ detected across our samples coincide with terminations. The boundary between PATI-9/8 and 8/7 occurred within MIS 8 and indicates an increase in the flux of organic carbon through that glacial MIS. This may reflect increasing efficiency of the plankton multiplier of Woods and Barkmann (1993).

The close grouping of the boundaries between PATI-5 through PATI-1, all of which occurred during the transition from MIS 2 to MIS 1, show this to have been an interval of protracted environmental change at ODP Site 994. Sea level rose by ∼120 m during termination T-1 [61], but did so in several decimeter steps [62]. It is possible that the closely spaced boundaries from PATI-5 through PATI-1 reflect these steps. *Brizalina lowmani* did not decrease in proportional abundance across all boundaries between PATI-5 through PATI-1, but increased across the PATI-3/2 boundary.

Some data suggest that the changes in the fauna across the peaks in values of ATI_s_ reflect changes in either (a) dissolved oxygen levels, (b) the organic carbon flux and (c) bottom current strength, although the first two of these factors are frequently correlated [63]. For a paleoenvironmental summary, see Table 3.

**Table 3:** Environmental interpretation of peak-bounded ATI_s_ (PATI-) intervals in the Upper Quaternary in ODP Hole 994C, Blake Ridge.

ATI interval	Isotope stage	Notable species presence/absence	Paleoenvironmental interpretation
PATI-1	MIS 1	Few *Melonis baarleeanus*, abundant *Cibicides wuellerstorfi*	decreased seasonality, low organic carbon flux, enhanced current action
PATI-2	? MIS 2/1	Few *Melonis baarleeanus*	decreased seasonality
PATI-3	? MIS 2/1	Few *Melonis baarleeanus*, abundant *Cibicides wuellerstorfi*	decreased seasonality, low organic carbon flux, enhanced current action
PATI-4	? MIS 2/1	Abundant *Melonis baarleeanus*	enhanced seasonality
PATI-5	MIS 4/3/2	Abundant *Melonis baarleeanus*	enhanced seasonality
PATI-6	MIS 6/5	Abundant *Cibicides wuellerstorfi*, *Epistominella exigua*	low organic carbon flux, enhanced current action and seasonality
PATI-7	MIS 8	Abundant *Uvigerina hispidocostata*	enhanced carbon flux
PATI-8	MIS 8	Abundant *Uvigerina hispidocostata*	enhanced carbon flux
PATI-9	MIS 9	Abundant *Cibicides wuellerstorfi*	low organic carbon flux, enhanced current action
PATI-10	MIS 11/10	Abundant *Melonis baarleeanus*	enhanced seasonality
PATI-11	MIS 12	Dominant *Bulimina aculeata*	organic flux >3 g C m^−2^ yr^−1^

den Dulk et al. [64] studied benthonic foraminifera under an upwelling system in the northern Arabian Sea. They recognised two groups of foraminifera:

1. Species that prefer high dissolved oxygen and low organic carbon levels: (*Bulimina striata, Gavelinopsis lobatula, Chilostomella oolina, Sphaeroidina bulloides, Cibicides ungerianus, Hyalina balthica, Hoeglundina elegans, Melonis barleeanus, Quinqueloculina* spp., *Globocassidulina subglobosa and Cassidulina carinata*;
2. Species preferring low dissolved oxygen and high organic carbon: *Bulimina exilis, Rotaliatinopsis semiinvoluta, Brizalina alata, B. pygmaea, Globobulimina spp., and Bulimina* sp.1.

The species of *Brizalina* and *Bulimina*, which dominate in ODP Hole 994C, are limited to group 2. de Rijk et al. [65] found that *Bulimina aculeata*, which dominated in PATI-11 (Figure 3B), lives primarily where the flux of organic carbon exceeds 3 g m^−2^ yr^−1^. *Melonis baarleeanus* (Figure 3E), although placed in group 1 by den Dulk et al. [64], was shown by Qvale and Van Weering [66] to prefer a fine-grained substrate with a relatively high organic carbon content [67]. Mackensen et al. [68] suggested that in the South Atlantic Ocean it prefers seasonally varying productivity. Taldenkova et al. [69] found this species to be more abundant in the upper bathyal Holocene of the Arctic Ocean than the latest Pleistocene, and ascribed it to a distal-river group of relatively deep-water species that thrive on slightly altered organic matter and is therefore restricted to areas with periodic delivery of organic matter. Murray [70] noted that M. *baarleeanus* has been recorded live in all oceans except the Indian Ocean. In ODP Hole 994C this species accounted for >0.02 of the CoBI_t_ across six of the ten PATI boundaries, and was abundant in the early part of PATI-10 and in PATI-5 and PATI-4. It was rare to absent in PATI-3 through PATI-1. This suggests that seasonality varied through the Late Quaternary at Blake Ridge. Unlike in the Arctic Ocean [69], at Blake Ridge seasonality was much reduced in the Holocene, after termination T-1.

*Globocassidulina subglobosa*, which is found throughout the Atlantic, Pacific and Southern Oceans (Murray, 2013), has been suggested to be an oxic indicator [71] that prefers an elevated mean organic carbon flux of 15 g m^−2^ yr^−1^ [72]. This species was abundant in PATI-4 (which equates to a brief episode in the glacial MIS 2). Smart and Gooday [73] examined trends in benthonic foraminiferal abundances along an organic enrichment gradient on the continental slope off North Carolina, eastern Atlantic Ocean. They found *Bulimina aculeata* and *Globocassidulina subglobosa* to be equally abundant at all sites, suggesting that these cannot be used as proxies for the organic flux. It is unclear, however, if *Globocassidulina obtusa* and *G. murrhina* have the same tolerances.

Kaiho [74] suggested that many of the species recovered from ODP Hole 994C are indicative of suboxic bottom waters, although he also suggested that *Cibicides wuellerstorfi* (figure 3C) and *Cibicidoides robertsonianus* are indicative of oxic water [75]. The abundance of *C. wuellerstorfi* was relatively high during PATI-6. Altenbach et al. [72] recorded the annual organic carbon flux levels best tolerated by some live benthonic foraminifera. Most species recorded by Altenbach et al. [72] that were also recovered from ODP Hole 994C lived under a flux rate of ∼2–6 g m^−2^ yr^−1^ (*Bulimina striata mexicana, C. robertsonianus*, *Pullenia bulloides, P. quinqueloba*). Schönfeld [71] recorded these four species as living infaunally within the sediment at a variety of depths down to 4.5 cm. However, Altenbach et al. [72] recorded live *C. wuellerstorfi* primarily at low organic carbon flux rates of 1.5–3 g m^−2^ yr^−1^ and *Oridorsalis umbonatus* at 2–3.5 g m^−2^ yr^−1^. *Cibicides wuellerstorfi* is an epiphytal species living on raised substrate particles that prefers active bottom currents [76], [77]. CoBI_t_ showed in ODP Hole 994C that *C. wuellerstorfi* was recovered primarily from PATI-9, 6, 3 and 1, during which the organic carbon flux may have been low, the strength of the Western Boundary Undercurrent enhanced, or both. Altenbach et al. [72] recovered *Hoeglundina elegans* mainly from areas with a flux rate of 4.5–15 g m^−2^ yr^−1^, although Schönfeld [71] suggested it to be an oxic indicator. The costate species *Uvigerina mediterranea* and *U. peregina*, which have morphologies comparable to *U. hispidocostata*, primarily under a carbon flux of 3–9 g m^−2^ yr^−2^. Schönfeld [71] recorded *U. peregrina* as living mostly at depths of 0–1 cm below the sediment water interface, but *U. mediterranea* as occurring down to 6 cm below the interface. *Uvigerina hispidocostata* in ODP Hole 994C was recovered mainly from PATI-8 and PATI-7, which coincide with MIS 8, for which it could indicate an interval of enhanced organic carbon flux but might also highlight an interlude in which uvigerinids penetrated deeper into the sediment. Schoenfeld and Altenbach [78] found that *Uvigerina* spp. in the north-eastern Atlantic Ocean were more abundant during glacial MIS 2 than interglacial MIS 1, and ascribed this to a widespread change from glacial to modern productivity characteristics across termination T-I. MIS 8 is similarly a glacial stage. Seiglie [79] noted *B. lowmani* to be indicative of high organic carbon levels and Sen Gupta and Strickert [80] found it to be dominant on the continental slope off Florida at depths >100 m, below the Gulf Stream. In ODP Hole 994C *B. lowmani*, *Cassidulina laevigata*, *Cibicides wuellerstorfi*, *Melonis baarleeanus*, *M. pompilioides* and *Uvigerina hispidocostata* had the highest number of PATI boundaries across which CoBI_p_ >0.02, while *B. lowmani*, *Cibicidoides robertsonianus*, *Globocassidulina obtusa*, *M. baarleeanus*, *M. pompilioides*, *U. celtica* and *U. hispidocostata* had the highest number PATI boundaries across which CoBI_t_ >0.02. These indicate that the organic carbon flux, dissolved oxygen levels and bottom current strength varied between PATIs, rather than between glacials and interglacials.

Gooday et al. [81] found *Epistominella exigua* (Figure 3D to be abundant in well-oxygenated, abyssal water below the oxygen minimum zone of the Arabian Sea. Smart et al. [82] showed that *E. exigua* colonises aggregates of phytodetritus and they speculated that this opportunistic, epifaunal species may represent a proxy for seasonal phytodetritus pulses originating from surface primary productivity in open ocean eutrophic areas. They suggested that inputs added over a geologically prolonged period of time would be reflected in peaks of *E. exigua*. This species was at its most abundant in PATI-6, having a ATI_p_ >0.05 across the PATI-7/6 boundary and a ATI_t_ >0.05 across both the PATI-7/6 and PATI-6/5 boundaries. This implies a brief interlude of enhanced seasonality in MIS 7 and 6, and may be related to a change in surface circulation and the position of the Gulf Stream at that time.

Sequence stratigraphy is the correlation of sedimentary rock successions using key events produced by worldwide changes in sea level [1]. These events are used to divide the succession into packages (systems tracts) that are bounded by characteristic surfaces [83], [84]. Benthonic foraminifera have long been used in sequence stratigraphy at neritic paleodepths [85]. However, the use of benthonic foraminiferal assemblage characteristics for sequence stratigraphic purposes at abyssal depths has thus far been problematic. For example, at neritic depths the planktonic/benthonic foraminiferal ratio has been used to determine changes in sea level [86], [87]. However, this index cannot be readily applied at depths of more than ∼500 m, at which planktonic foraminifera typically form >99% of the assemblage. At neritic depths, maximum flooding surfaces are reflected by peaks in uvigerinid abundance that have been ascribed to sluggish circulation at times of maximum transgression [88]. This is contrary, however, to the enhanced abundance of bathyal and abyssal *Uvigerina* during glacial lowstands. Nagy et al. [89] suggested that at neritic depths the information function H is low on interglacial maximum flooding surfaces. In ODP Hole 994C, however, H is negatively correlated with δ18O and the index is lower during glacial, even numbered MIS than it is during interglacial MIS.

Therefore, we propose that ATI_s_ peaks show strong potential as a sequence stratigraphic tool for abyssal deposits, some peaks at the PATI boundaries coinciding with terminations that are marked by transgressive systems tracts. However, the apparent coincidence between peaks in ATI_s_ and terminations must be applied with caution, since not all peaks coincide with terminations; two peaks occurred within glacial MIS 8. However, this can be avoided by judicious use of the control limits.

## Conclusions

Assemblages are not constant entities, but change over time as the proportional abundance of each species within a community changes. As one species acquires a higher proportional abundance, one or more others must decrease in abundance. Peaks in ATI_s_, the ATI between successive samples, delimit peak-bounded intervals (PATIs-) within which the community is relatively stable. The current inability to detect glacial-interglacial contrasts in general appears to arise because not all sites show marked changes in community composition at the species level at glacial-interglacial boundaries. Both ATI and CoBI can be applied to successions for which there is no immediately obvious differentiation of glacial and interglacial assemblages. Peaks in ATI_s_ in the Upper Quaternary of ODP Hole 994C, Blake Ridge, define eleven PATIs. Eight of the PATI boundaries approximate to terminations, although, as shown by termination T-I, a termination can be marked by more than one PATI boundary if, like termination T-I, it consists of a series of events marked by decimeter changes in sea level. While it appears that for our data set all terminations were marked by at least one PATI boundary, not all PATI boundaries coincided with terminations; two PATI boundaries were recorded within MIS 8. Nevertheless, this suggests that PATIs and peaks in assemblage turnover as measured by our index, ATI_s_, have potential as a sequence stratigraphic tool. Our quantitative approach allows some sequence stratigraphic concepts to be extended into the abyssal environment.

Both CoBI_p_ and CoBI_t_ suggest that species that changed markedly across PATI boundaries were responding to changes in paleo-oxygenation, the organic matter flux, or bottom current strength. A transitory peak in *Epistominella exigua* within PATI-6 implies a brief interlude of enhanced seasonality in MIS 7 and 6, and may be related to a change in surface circulation and the position of the Gulf Stream at that time.

The assemblage turnover (ATI) and conditioned-on-boundary (CoBI) indices have here been applied to the ecostratigraphy of abyssal benthonic foraminifera. However, these measures can also be used to detect and characterise boundaries for any taxon and applied in both paleoecological and ecological studies.

## Supporting Information

Appendix S1 **Terms introduced in this paper and their definitions.**
(DOCX)

File S1 **Data repository.** Benthonic foraminifera in the Upper Quaternary of ODP Hole 994C.
(XLSX)
