# Supplementary material for: Quantifying Assemblage Turnover and Species Contributions at Ecologic Boundaries
Source: PLoS One. 2013 Oct 9;8(10):e74999. doi: 10.1371/journal.pone.0074999 (PMC3794043; doi:10.1371/journal.pone.0074999)
Supplement: Appendix S1 — Terms introduced in this paper and their definitions. (DOCX) [file pone.0074999.s001.docx]

APPENDIX S1: TERMS INTRODUCED IN THIS PAPER AND THEIR DEFINITIONS

ATI: Assemblage Turnover Index: a measure of the extent to which the proportional or percentage abundances of species change between two units (whether samples or PATIs) along a transect. The transect may be either stratigraphic, as in this study, or biogeographic.

ATI_s_: assemblage turnover between a pair of samples, calculated as ATI_s_ = ∑│p_i2_-p_i1_│, where p_i1_ and p_i2_ are the proportional abundances of the *i*th species, i=1,2…,s, in the lower (older) and upper (younger) sample respectively.

PATI: the stratigraphic interval between two maximal peaks in ATI_s_. In this study a peak in ATI_s_ is defined as occurring where the value of ATI_s_ exceeds the mean plus one standard deviation. Thus, two adjacent samples for which ATI_s_>(+ σ) will form separate peaks.

ATI_p_ (Partial Assemblage Turnover): assemblage turnover between all samples in an entire PATI and the single sample immediately overlying the peak in ATI_s_ that forms the upper boundary of the PATI. It is calculated from the same formula as ATI_s_ but designated as ATI_p_ = ∑│p_i2_-p_i1_│, to indicate that p_i1_ is the proportional abundance of the *i*th species in a PATI and p_i2_ is the proportional abundance of the same species in the sample succeeding the peak in ATI_s_ that forms the upper boundary of the PATI. This is a quantitative measure of the short-term assemblage change exactly at the boundary of a PATI.

CoBI: Conditioned-on-Boundary Index, which measures the contribution each species makes to the total turnover. This is always conditional on or conditioned-on a particular boundary. That is, the measure is relevant for examining only a single particular selected boundary of interest.

CoBI_p_ (Partial Conditioned-on-Boundary Index): a measure of the proportional contribution of each species to ATI_p_. For each species this measure is calculated as CoBI_p_ = (│p_i2_-p_i1_│)/ ATI_p_ where p_i1_ is the proportional abundance of the *i*th species in a PATI and p_i2_ is the proportional abundance of the same species in the sample succeeding the peak in ATI_s_ , which forms the upper boundary of the PATI.

ATI_t_ (Thorough Assemblage Turnover): the total assemblage turnover between two adjacent PATIs separated by a peak in ATI_s_. It is calculated from the same formula as that for ATI_s_, but denoted by ATI_t_ = ∑│p_i2_-p_i1_│, to indicate that p_i1_ is the proportional abundance of the *i*th species in the lower (older) PATI and p_i2_ is the proportional abundance of the same species in overlying PATI. This is a quantitative measure of the difference of the two assemblages or the turnover that exists between two PATIs.

CoBI_p_ (Thorough Conditioned-on-Boundary Index): a measure of the proportional contribution of each species to ATI_t_. For each species this measure is calculated as CoBI_t_ = (│p_i2_-p_i1_│)/ ATI_t_ where p_i1_ and p_i2_ is the proportional abundance of the *i*th species in each of the successive PATIs.
